# Driving Sustainable Popcorn Breeding for Drought Tolerance in Brazil

**DOI:** 10.3389/fpls.2021.732285

**Published:** 2021-09-21

**Authors:** Samuel Henrique Kamphorst, Antônio Teixeira do Amaral Junior, Valter Jário de Lima, Marcelo Javier Carena, Valdinei Cruz Azeredo, Gabrielle Sousa Mafra, Pedro Henrique Araújo Diniz Santos, Jhean Torres Leite, Kátia Fabiane Medeiros Schmitt, Divino Rosa dos Santos Junior, Rosimeire Barboza Bispo, Talles de Oliveira Santos, Uéliton Alves de Oliveira, Jacymara Lopes Pereira, Danielle Leal Lamêgo, Carolina Macedo Carvalho, Letícia Peixoto Gomes, José Gabriel de Souza Silva, Eliemar Campostrini

**Affiliations:** ^1^Laboratório de Melhoramento Genético Vegetal, Centro de Ciências e Tecnologias Agropecuárias (CCTA), Universidade Estadual do Norte Fluminense Darcy Ribeiro - UENF, Campos dos Goytacazes, Brazil; ^2^AgResearch Ltd, Grasslands Research Centre, Palmerston North, New Zealand

**Keywords:** gene effects, combining ability, food security, grain yield, popping expansion

## Abstract

Drought currently affects several regions worldwide and tends to be more frequent due to climate change. It might compromise food security and the economic structure related to agribusiness. Popcorn has a crucial role in the Brazilian economy, but the cultivars that adapt to water stress, the most prejudicial abiotic stress for crop productivity, are unknown to date. This deficit of popcorn varieties adapted to heat and drought stresses will become more limiting with climate change. Given this scenario, knowing the genetic basis of agronomic traits under stress conditions is essential in promoting crop productivity and plant adaptation to abiotic stresses. Under two contrasting water conditions (WC) and different crop seasons (CS), we aimed to generate information about the combining ability of 10 popcorn progenitors and 15 hybrids through a partial diallel-mating design. The water stress was initiated at the male pre-anthesis stage. Significant genotype^*^crop seasons (G^*^CS), genotype^*^water condition (G^*^WC), and genotype^*^crop seasons^*^water condition (G^*^CS^*^WC) interactions were present. Regardless of CS and WC, non-additive effects controlled grain yield (GY), grain number per row (GN), ear length and diameter (ED), and 100-grain weight, while additive effects were present for popping expansion (PE). For each CS, regardless of WC, the cause-effect of GN (2018) and ED (2020) on GY seems to be an opportunity for indirect selection. Utilizing genetically broad-based hybrids is also a good opportunity for obtaining superior genotypes for GY and PE as it is possible to select inbred lines for both of these traits. We recommend the L76 × L61 hybrid for the Brazilian agribusiness context due to its greater productivity and dominance deviations.

## Introduction

Drought is the main environmental stress causing losses in agricultural productivity worldwide (Araus et al., 2010; Adebayo et al., 2014; Daryanto et al., 2016; Fahad et al., 2017). The losses resulting from water restriction has been predicted to become more frequent (Awange et al., 2016; Van Loon et al., 2016), and diminishing its effects has been a challenge for agribusinesses on a global scale and in Brazil, in particular, where the economy is heavily dependent on agricultural activities. Due to the harmful effects caused by drought on crop growth and development, plant breeding has been used as a powerful tool to reduce the vulnerability of crops (Challinor et al., 2016; Araus et al., 2018). Breeders have led research to the understanding of the morphological, physiological, and agronomic responses of plants under water stress and the selection of more productive genotypes under limiting conditions (Araus et al., 2010; Adebayo et al., 2014; Altieri and Nicholls, 2017; Dias et al., 2018; Kamphorst et al., 2019, 2020a,b; Lima et al., 2019). Tjis was done with the objective of mitigating the impacts of climate change mainly caused by irregular precipitation (Cunha et al., 2019; Malhi et al., 2021). Popcorn has a crucial role in the economy of the USA where its sales are responsible for ca. one billion dollars annually, and there is also a high demand for this product in Brazil (Carvalho et al., 2020). As such, traditional maize has several examples of integrating pre-breeding with cultivar development for drought tolerance (Carena et al., 2009; Carena, 2011, 2013, 2021; Sharma and Carena, 2016). However, there is a deficit of popcorn cultivars adapted to soil water stress, although the first steps have already been taken (Lima et al., 2019; Kamphorst et al., 2020b, 2021; Santos et al., 2020; Leite et al., 2021).

Historically, plant breeding has been used to develop cultivars for optimal environmental conditions. Water- and nutrient-use efficiency and/or tolerance have never been targeted before for selection, given that the experimental stations of seed companies do not experience these limitations (Carena, 2021). However, the scenario is different in Brazilian farmlands where no expensive and efficient irrigation systems can be found (Carvalho et al., 2020; Zilli et al., 2020). Erratic rainfall has been frequently reported in the last decades (Intergovernmental Panel on Climate Change, 2019) and, hence, exacerbates these soil water deficits. Thus, under soil water stress, popcorn cultivars cannot express their maximum genetic potential.

Drought causes physiological and biochemical disturbances in plants, which reduce leaf expansion and cellular metabolic activities. This, on turn, leads to stomatal closure and reductions in the photosynthetic CO_2_ assimilation rate, consequently causing changes in the carbon partition throughout the plant and, therefore, lower crop productivity (Lopes et al., 2011; Dalal and Sharma, 2017; Blankenagel et al., 2018). Drought impacts on crop productivity may also vary depending on the level of sensitivity, intensity, and duration of the stress genotype and the stage of plant development when the stresses occur (Cairns et al., 2012). In particular, the critical stage of drought occurrence in maize extends from flowering to grain filling (Liu and Qin, 2021).

Understanding the genetic basis related to agronomic traits of interest is extremely relevant to plant breeding programs, which aim to increase crop productivity in drier areas through attempts to generate superior hybrid and segregants (Cruz et al., 2014). In this context, the diallel-mating design has been widely used in several cultivated species to provide genetic information through estimations of the combining ability of progenitors and their hybrids (Hallauer et al., 2010; Cruz et al., 2014). Through mating designs, it is possible to estimate the existence of the additive effects of the progenitors and the non-additive ones at the crosses. Additionally, these kinds of designs are also appropriate for the estimation of the relative importance of the additive, non-additive, and environmental effects on plant traits (Cruz et al., 2012). However, a drawback to the diallel-mating design is the number of crosses needed when compared with other mating designs (e.g., design I or II). To combat this, a greater flexibility over crosses can be obtained through the utilization of a partial or circulating diallel as proposed by Kempthorne and Curnow (1961), consequently reducing the number of hybrids produced. Furthermore, basic genetic information obtained either by a complete diallel analysis or circulating diallel about the mechanism of action of the main popcorn traits, e.g., grain yield (GY) and popping expansion (PE), under water stress conditions is scarce (Lima et al., 2019). The understanding of these genetic mechanisms might open up opportunities for increasing popcorn productivity and plant adaptation to water stresses.

Genetic effects controlling popcorn traits under well-watered conditions (Larish and Brewbaker, 1999; Pereira and Amaral Júnior, 2001; Silva et al., 2010; Jele et al., 2014; Dar et al., 2018; Coan et al., 2019; Schwantes et al., 2020), biotic stresses (Schwantes et al., 2017; Mafra et al., 2018; Amaral Júnior et al., 2019; Kurosawa et al., 2020, 2021), and nutritional deficiencies (Gerhardt et al., 2019; Santos et al., 2019, 2020) have revealed that the additive genetic action is prevalent for PE, whereas the non-additive action has been the most important for GY expression and its components. In addition, non-additive genetic effects were the most prevalent controlling GY under water limitations in tropical maize (Betrán et al., 2003). The greater influence of non-additive genetic effects related to GY under drought conditions was also observed by Derera et al. (2008) and Dhliwayo et al. (2009). Edmeades et al. (1999) supported these observations by reporting additive gene action in response to selection based on GY for thre tropical maize populations under water stress.

To fill the current gaps in knowledge related to the genetic control on popcorn traits of economic interest under drought conditions, we asked the following questions: What is the main genetic action underlying popcorn trait expression (GY and PE) under drought? Is the genetic action the same under water stress and well-watered conditions? What plant breeding methods can be recommended under both conditions? We aimed to generate information on the combining ability of 10 popcorn progenitors and selected hybrids evaluated under water stress and well-watered conditions in different crop seasons (2018 and 2020). Our main goal was to propose popcorn breeding strategies focused on the development of more cultivars adapted to water stress for the Brazilian agribusiness context.

## Materials and Methods

### Plant Material, Site Description, and Growing Conditions

This study evaluated 10 popcorn inbred lines (S_7_) as progenitors and 15 hybrids. We selected S_7_ inbred lines from 20 inbred lines previously tested under water limiting conditions (Kamphorst et al., 2018a, 2020a). After a performance evaluation, we selected four inbred lines and classified them as agronomic water use efficient (P2, P3, P6, and P7), four as inefficient (L61, L63, L65, and L75), and two as intermediate (L71 and L76; Kamphorst et al., 2018a). Inbred lines L61, L63, L65, L71, and L75 were derived from the population “BRS-Angela,” which was adapted to tropical climates; P6 and P7 were derived from the commercial hybrid “Zélia”; P2 and P3 were derived from compound CMS-42; L76 was derived from the population “Viçosa,” which was adapted to temperate and tropical climates (Vittorazzi et al., 2018).

Hybrids were obtained following the methodology proposed by Kempthorne and Curnow (1961). We considered the number of *s* (crosses) equal to three (Table 1).

**Table 1:** Hybrid combinations from circulating diallel across 10 popcorn inbred lines with crosses (*s*) = 3.

**Inbred lines**	**L76**	**P3**	**P6**	**L63**	**P7**	**L61**	**L71**	**P2**	**L65**	**L75**
L76					X	X	X			
P3						X	X	X		
P6							X	X	X	
L63								X	X	X
P7									X	X
L61										X
L71										
P2										
L65										
L75										

Inbred lines were crossed in pairs, considering that the 6-m planting rows should be spaced 1 m apart from each other and that there should be 0.4-m spacing between plants. The ears of each inbred line were covered with shoot bags before anthesis. Then, mature tassels from the opposite row were covered with tassel bags. Pollination was done manually and controlled over 65 days after planting. We obtained the seeds of each hybrid in April 2017 from the Experimental Station of the Antônio Sarlo Agricultural State College in Campos dos Goytacazes, Brazil (21 42′48″S, 41 20′38″W; 14 m asl).

### Experimental Design and Cultural Treatment

The experiments were carried out at the same Experimental Station (Antônio Sarlo Agricultural State College) during the dry seasons (CS) of 2018 and 2020 (April and August).

The experimental design was arranged in completely randomized blocks with three repetitions for both well-watered (WW) and water stress (WS) conditions. The plots comprised two 4.4-m rows, spaced 0.2 m between plants and 0.8 m between rows (44 plants per plot representing 62,500 plants ha^−1^). Planting fertilization included 30 kg ha^−1^ N (urea), 60 kg ha^−1^, P_2_O_5_ (triple superphosphate), and 60 kg ha^−1^ K_2_O (potassium chloride), with a side dress application (30 days after sowing) of 100 kg ha^−1^ N (urea).

The plants were irrigated through a drip system, specifically using a Katif dripper for each plant at a flow rate of 2.3 mm h^−1^. The WW condition received irrigation at soil-field capacity (−0.01 MPa). Soil water status was monitored using three Decagon MPS-6 sensors (Decagon Devices, Inc., Pullman, WA, USA) installed at a depth of 0.2 m in the planting line between two plants. Soil water status under WS conditions was also monitored with the same sensors, and the irrigation was withheld ≈15 days before male anthesis (June 22nd to June 26th during both CS).

During the experimental period, the total rainfall was 148.2 and 117 mm in 2018 and 2020, respectively (Table 2). Under WS conditions, the plants received 69.3 and 52.3 mm of water and, under WW conditions, 187.8 and 137.8 mm during CS 2018 and 2020, respectively (Table 2).

**Table 2:** Precipitation and irrigation (mm) applied in experiments under well-watered (WW) and water-stressed (WS) conditions in the crop seasons of 2018 and 2020.

	**2018**					**2020**				
**Weeks after sowing**	**Precipitation (mm)**	**WW (mm)**		**WS (mm)**		**Precipitation (mm)**	**WW (mm)**		**WS (mm)**	
		**Irrigation**	**Total**	**Irrigation**	**Total**		**Irrigation**	**Total**	**Irrigation**	**Total**
1	17	7.2	24.2	6.2	23.2	2	7.2	9.2	6.9	8.9
2	6	11	17	10.2	16.2	6.2	27.1	33.3	20.2	26.4
3	0	10.1	10.1	9.9	9.9	24.6	3.7	28.3	3.6	28.2
4	10.6	10.7	21.3	10.3	20.9	12.6	3.9	16.5	1	13.6
5	5.2	8.4	13.6	8.4	13.6	30	7	37	4.3	34.3
6	2	11.6	13.6	12.2	14.2	0.6	8.1	8.7	10.8	11.4
7	0	12.9	12.9	12.1	12.1	0.6	5.4	6	5.4	6
8	0	10.9	10.9	0	0	5.4	4.7	10.1	0	5.4
9	0	18.8	18.8	0	0	1.6	13.7	15.3	0	1.6
10	0	18.9	18.9	0	0	2.2	15	17.2	0	2.2
11	30.8	1.1	31.9	0	30.8	8.2	5.5	13.7	0	8.2
12	0	16.7	16.7	0	0	1.2	7.3	8.5	0	1.2
13	0	14	14	0	0	0.4	7.7	8.1	0	0.4
14	65	2	67	0	65	0.8	15.1	15.9	0	0.8
15	0	13.5	13.5	0	0	20.4	2.4	22.8	0	20.4
16	9.2	10	19.2	0	9.2	0.2	4.3	4.5	0	0.2
17	2.4	10	12.4	0	2.4	0	0	0	0	0
Total	148.2	187.8	336	69.3	217.5	117	137.8	254.8	52.3	169.3

In CS 2018, the soil under WS reached their permanent wilting points (ψ_s_ > −1.5 Mpa) 70 and 93 days after sowing, which were during the flowering and grain-filling stages, respectively (Supplementary Figure 1). In CS 2020, two negative peaks for water potential at 69 and 85 days after sowing (Supplementary Figure 1) were recorded. Rainfall episodes in CS 2018 also led soil to field water capacity; however, in CS 2020, even with rainfall episodes, soil remained below ψ_s_-1.0 Mpa (Supplementary Figure 1).

During the crop cycle in the 2018 and 2020 harvests, the average temperature was 21.59 and 21.76°C and the relative humidity was 78.45 and 76.85%, respectively. The average solar radiation was ≅ 1,189 and 1,240 μmol m^−2^ s^−1^ in CS 2018 and 2020, respectively (Supplementary Figure 2). Weather conditions were recorded at a National Institute of Meteorology (INMET) weather station located near the experimental site (21 42′48″S, 41 20′38″W; 14 m asl).

### Traits Evaluated

Grain yield was obtained after threshing the ears of each plot and then corrected with 13% humidity (kg ha^−1^). Popping expansion was measured by the mass of 30 g of grains placed in a microwave oven in a kraft bag for 2 min, with the volume of the popcorn quantified in a 2,000-ml beaker and the ratio of the popped volume being divided by 30 g and expressed in ml g^−1^. Grain number per row (GN) was determined by counting, ear diameter (ED) was estimated with a Vernier caliper (mm), and ear length (EL) was measured with a ruler (cm). To determine the 100-grain weight (GW), we weighed (g) two samples of 100 grains per plot. The traits GW, PE, and GY were then measured in all the plants of each plot, while GN, ED, and EL were measured using one random sample of six plants per plot.

### Statistical Analyses

The genetic-statistical and path analyses were performed using the GENES software (Cruz, 2013).

#### Analysis of Variance

The experiments were analyzed considering the following mathematical model: y_ijk_ = μ + (b/c)/wc_jkm_ + g_i_ + cs_j_ + w_k_ + gcs_ij_ + gwc_ik_ + cswc_jk_ + gcswc_ijk_ + e_ijk_, where the mean (μ), the effect of genotype (g), crop (cs), water condition (wc), G × CS interaction (gcs), G × WC interaction (gwc), CS × WC interaction (cswc), and G × CS × WC (gcswc) were considered fixed and the block within WC within CS [(b/cs)/w] and error (e) were considered random.

When the G^*^WC interaction was significant, we performed an ANOVA for each WC considering the linear model Y_ij_ = μ + g_i_ + b_j_ + e_ij_, where mean (μ) and genotype effect (g) were considered fixed, and block (b) and error (e) were considered random.

#### Diallel Analysis by Kempthorne and Curnow

In each CS, we performed the circulating diallel analysis as follows: Y_ij_ = μ + g_i_ + g_j_ + s_ij_ + wc_k_ + gwc_ik_ + gwc_jk_ + swc_ijk_ + e_ij_, where: Y_ij_ = mean observation associated with the hybrid combination ij (i ≠ j), or with parent *I* (*I* = *j*); μ = general mean; g_i_ and g_j_ = general combining ability effects associated with progenitors *i* and *j*, respectively; s_ij_ = specific combining ability effects associated with progenitors *i* and *j*; w_k_ = effect of water condition *k*; g_lik_ and g_ljk_ = general combining ability effects associated with progenitors *i* and *j* and water condition *k*, respectively; s_lijk_ = specific combining ability effects associated with progenitors *i* and *j* and water condition *k*; e_ij_ = mean experimental error.

When significant positive interactions with crop season (CS – 2018 and 2020) were observed, we performed individual analyses through the following genetic-statistical model: Y_ij_ = μ + g_i_ + g_j_ + s_ij_ + e_ij_, where Y_ij_ = mean observation associated with the hybrid combination ij (i ≠ j), or with parent *i*(*i* = *j*); μ = general mean; g_i_ and g_j_ = general combining ability effects associated with progenitors *i* and *j*, respectively; s_ij_ = specific combining ability effects associated with progenitors *i* and *j*; e_ij_ = mean experimental error. We considered water condition and its interaction as fixed effects.

#### Path Analysis

The values of each trait per plot were standardized $(y_{i}^{\prime }=\frac{y_{i}-\bar{y}S_{y}}{)}$ and then subjected to an ANOVA to generate the genotype correlation matrix. Then, we obtained the genotypic correlation estimates (*r*_*g*_) according to Mode and Robinson (1959). The significance of *r*_*g*_ was tested by *t*-tests, at 5 and 1% probability, with *n* – 2 degrees of freedom between all pairs of combinations. The genotype correlation matrix was submitted to a collinearity test according to Christensen and Montgomery (1981).

Genotype correlation was then used to estimate the path coefficients, partitioning them into direct (p_jy_) and indirect (r_ij_p_jy_) effects by estimating the regression equation under both WCs (WW and WS) according to the model of primary traits (GN, EL, ED, and GW), which explained the variation of Y (GY) and PE. Thus, r_ij_ is the correlation coefficient of trait i with j, and p_jy_ was the direct effect (or path coefficient) of trait j in the final product Y.

The resolution as a matrix was obtained by the normal equation system X'Xβ = X'Y, where X'X is a non-singular matrix of correlations between explanatory variables, β is a column-vector of path coefficients, and X'Y is a column-vector of correlations between the main and explanatory variables.

The coefficient of determination for the analysis of explanatory variables on the main variable was given by: $R_{0.123\ldots n}^{2}={\hat{p}}_{01}r_{01}+{\hat{p}}_{02}r_{02}+{\hat{p}}_{03}r_{03}+$. + ${\hat{p}}_{05}r_{05}$. The residual effect was expressed by: $\text{p}\varepsilon ={\left(1-R_{0.123\ldots n}^{2}\right)}^{.5}$.

## Results

### Genetic Variability for Agronomic Traits Evaluated in Popcorn Under Different WC and CS

The genotype and water condition (WC) sources of variations differed statistically for all agronomic traits (Table 3). For the sources of variation of the CSs, GY, GN, EL, ED, and 100-GW differed statistically except for PE (Table 3).

**Table 3:** Summary of the analysis of variance, mean values, and coefficient of variation (CV) of agronomic traits evaluated in popcorn genotypes under different water conditions (WC) and crop seasons (CS).

**SV**	**G^#^**	**CS^#^**	**WC^#^**	G*CS^#^	G*WC^#^	CS*WC^#^	G*CS*WC^#^	**Residual^#^**	$\bar{\text{X}}$	**CV (%)**
	**DF = 24**	**DF = 1**	**DF = 1**	**DF = 24**	**DF = 24**	**DF = 1**	**DF = 24**	**DF = 192**		
GY	9^**^	2.19^*^	139.43^**^	0.57^**^	0.9^**^	5.77^**^	0.26^**^	0.10	2.13	15.14
PE	155^**^	25.17 ^ns^	99.07^**^	19.85^**^	10.52^*^	8.53^ns^	10.81^*^	6.35	25.13	10.03
GN	351^**^	1844.43^**^	1889.23^**^	19.51^ns^	8.51^ns^	270.52^**^	16.04^ns^	13.37	23.56	15.52
EL	26^**^	36.76^**^	117.1^**^	2.75^**^	0.79^ns^	10.29^ns^	1.16^ns^	1.32	11.75	9.78
ED	83^**^	808.52^**^	816.29^**^	5.93^*^	4.95^ns^	8.26^ns^	2.58 ^ns^	3.52	30.91	6.07
GW	17^**^	69.62^**^	218.42^**^	3.14^**^	1.23^ns^	28.57^**^	1.18^ns^	1.37	14.19	8.26

*SV, sources of variation; G, genotypes; CS, crop seasons; WC, water condition; DF, degrees of freedom*;

# *Mean squares*; $\bar{X}$, *average; CV, coefficient of variation experimental; GY, grain yield (t ha^−1^); PE, popping expansion (g ml^−1^); GN, grain number per row (unit); EL, ear length (cm); ED, ear diameter (mm); GW, 100-grain weight (g)*.

ns *Not significant at a 5% probability level in the F-test*.

*,** *Significant at 5 and 1% probability levels in the F-test, respectively*.

In general, traits showed significant interactions between G^*^C) except for GN (Table 3). The significant interaction between G^*^WC occurred in GY and PE, and significant interaction in CS^*^WC was observed for PE, EL, and ED (Table 3).

The interaction G^*^CS^*^WC was only observed for GY and PE traits (Table 3).

### GCA and SCA Combined in Different CS and WC for Agronomic Traits Evaluated in Popcorn

Significant differences were observed for the general (GCA) and specific combining ability (SCA) sources of variation during CS 2018 and 2020 (Table 4).

**Table 4:** Mean square estimates of popcorn genotypes for GCA and SCA and the residual and mean squares of combining ability effects on agronomic traits in distinct water conditions (WCs) and crop seasons (CSs).

**SV**	**GCA** ^**#**^		**SCA** ^**#**^		GCA*WC ^**#**^		SCA*WC ^**#**^		**Residual** ^**#**^	
	**DF = 9**		**DF = 15**		**DF = 9**		**DF = 15**		**DF = 96**	
**Traits / CS**	**2018**	**2020**	**2018**	**2020**	**2018**	**2020**	**2018**	**2020**	**2018**	**2020**
GY	3.25^**^	1.78^**^	4.71^**^	7.56^**^	0.40^**^	0.33^**^	0.37^**^	1.05^**^	0.11	0.10
PE	231.01^**^	129.46^**^	45.55^**^	18.48^**^	3.42^ns^	9.13^ns^	12.73^**^	13.85^*^	5.57	7.13
GN	176.96^**^	242.84^**^	144.85^**^	196.00^**^	12.40^ns^	5.02^ns^	23.33^ns^	5.49^ns^	16.08	10.66
EL	7.17^**^	10.80^**^	15.08^**^	20.03^**^	0.94^ns^	0.60^ns^	1.52^ns^	0.67^ns^	1.03	1.61
ED	30.61^**^	28.04^**^	45.49^**^	61.48^**^	4.25^ns^	5.83^ns^	2.38^ns^	3.63^ns^	2.98	4.06
GW	12.43^**^	7.29^**^	10.98^**^	9.75^**^	1.26^ns^	1.26^ns^	1.47^ns^	0.87^ns^	1.38	1.37
**Traits/CS**	**2018**					**2020**				
	**X**	${\hat{\phi }}^{2}g$	${\hat{\phi }}^{2}s$	${\hat{\phi }}^{2}g\text{w}c$	${\hat{\phi }}^{2}s\text{w}c$	**X**	${\hat{\phi }}^{2}g$	${\hat{\phi }}^{2}s$	${\hat{\phi }}^{2}g\text{w}c$	${\hat{\phi }}^{2}s\text{w}c$
GY	2.22	0.13	0.77	0.02	0.09	2.05	0.07	1.24	0.02	0.32
PE	24.84	9.39	6.66	−0.18	2.39	25.42	5.10	1.89	0.17	2.24
GN	21.08	6.70	21.46	−0.31	2.42	26.04	9.67	30.89	−0.47	−1.72
EL	11.41	0.26	2.34	−0.01	0.16	12.10	0.38	3.07	−0.08	−0.31
ED	29.27	1.15	7.08	0.11	−0.20	32.55	1.00	9.57	0.15	−0.14
GW	14.67	0.46	1.60	−0.01	0.03	13.70	0.25	1.40	−0.01	−0.16

*SV, sources of variation; GCA, general combining ability; SCA, specific combining ability; WC, water condition; DF, degrees of freedom*;

# *mean squares; X, average; GY, grain yield (t ha^−1^); PE, popping expansion (g ml^−1^); GN, grain number per row (unit); EL, ear length (cm); ED, ear diameter (mm); GW, 100-grain weight (g)*.

ns *Not significant at a 5% probability level in the F-test*.

*,** *Significant at 5 and 1% probability levels in the F-test, respectively*.

${\hat{\phi }}^{2}g$, *quadratic component associated with GCA*; ${\hat{\phi }}^{2}s$, *quadratic component associated with SCA*; ${\hat{\phi }}^{2}gwc$, quadratic component associated with GCA^*^WC; and${\hat{\phi }}^{2}swc$, quadratic component associated with SCA^*^WC.

Grain yield showed significant interactions for GCA^*^WC and SCA^*^WC in CS 2018 and 2020 (Table 4). The magnitude of the quadratic components highlighted the greatest importance of the SCA effects $({\hat{\phi }}^{2}s$) over GCA $({\hat{\phi }}^{2}g$) and the interaction of WCs with SCA^*^WC $({\hat{\phi }}^{2}swc$) and GCA^*^WC $({\hat{\phi }}^{2}gwc$) effects (Table 4). The additive effects $({\hat{\phi }}^{2}g$) explained 76 (2018) and 75% (2020) of the total variation for GY. Although GY showed significant interactions with GCA, the magnitude of the quadratic components of ${\hat{\phi }}^{2}swc$ effect was lower than 2% of the variation regardless of CS. Nonetheless, ${\hat{\phi }}^{2}swc$ effects varied from 9 (2018) to 19% (2020), which were greater than the magnitude of ${\hat{\phi }}^{2}g$ in 2020.

We observed a significant interaction between SCA^*^WC only for CS in both 2018 and 2020 (Table 4). Across different CS, the magnitude of the quadratic components showed greater importance for the GCA $({\hat{\phi }}^{2}g$) effects compared to SCA $({\hat{\phi }}^{2}s$) along with the interactions among the WC, SCA^*^WC $({\hat{\phi }}^{2}swc$), and GCA^*^WC $({\hat{\phi }}^{2}gwc$) effects (Table 4). The additive effects $({\hat{\phi }}^{2}g$) displayed 51 (2018) and 54% (2020) of the total variation in PE. We also observed that the quadratic component had a negative value (−0.18) for ${\hat{\phi }}^{2}gwc$ (Table 4). Only the effects of the interaction with ${\hat{\phi }}^{2}swc$ were significant for PE. This estimation accounted for 13 (2018) and 24% (2020) of the total variation in PE, with this effect being larger than the ${\hat{\phi }}^{2}s$ in 2020. Despite the magnitude of the effects of dominance showing lower values than the ${\hat{\phi }}^{2}swc$ in 2020, the estimation of the ${\hat{\phi }}^{2}s$ effects was responsible for 36 (2018) and 20% (2020) of the PE fluctuation.

Grain number, EL, ED, and GW did not express significant interactions between GCA^*^WC and SCA^*^WC across CS (Table 4). Therefore, for these traits, the magnitude of the quadratic components showed the greater importance of SCA $({\hat{\phi }}^{2}s$) effects over GCA $({\hat{\phi }}^{2}g$) across CS. The effects of ${\hat{\phi }}^{2}gwc$ and ${\hat{\phi }}^{2}swc$ were also null. Furthermore, the negative values observed for ${\hat{\phi }}^{2}gwc$ and ${\hat{\phi }}^{2}swc$ in the CSs of 2018 and 2020 in relation to GN, EL, ED, and GW should be considered real value estimates equal to zero (Table 4).

### Quadratic Components of GCA and SCA Individual in Different CS and WC for Agronomic Traits Evaluated in Popcorn

All the evaluated traits were significantly different for the GCA and SCA in both CSs, except PE for SCA in WW-2020 (Table 5). Regardless of CS and WC, the magnitude of the quadratic components of the GY, GN, EL, ED, and GW traits showed greater importance to the SCA $({\hat{\phi }}^{2}s$) effects over the GCA $({\hat{\phi }}^{2}g$) effects. Of all the traits, PE was the only one that displayed a greater importance to the GCA $({\hat{\phi }}^{2}g$) effects (Table 5).

**Table 5:** Mean squares estimates of popcorn genotypes for GCA and SCA and the residual and mean squares of combining ability effects on agronomic traits in distinct water conditions (WCs) and crop seasons (CSs).

	**2018**											
**SV**	**WS**						**WW**					
	**GCA** **(DF = 9)**	**SCA** **(DF = 15)**	**Residual** **(DF = 48)**	**X**	${\hat{\phi }}^{2}g$	${\hat{\phi }}^{2}s$	**GCA** **(DF = 9)**	**SCA** **(DF = 15)**	**Residual** **(DF = 48)**	**X**	${\hat{\phi }}^{2}g$	${\hat{\phi }}^{2}s$
GY^#^	1.20^**^	1.83^**^	0.13	1.68	0.09	0.57	2.44^**^	3.25^**^	0.08	2.76	0.20	1.06
PE^#^	129.65^**^	29.99^**^	5.29	24.43	10.36	8.23	104.78^**^	28.29^**^	5.85	25.24	8.24	7.48
GN^#^	86.02^**^	49.74^**^	17.35	17.62	5.72	10.80	103.35^**^	118.44^**^	14.80	24.54	7.38	34.55
EL^#^	3.20^*^	6.81^**^	1.18	10.59	0.17	1.88	4.92^**^	9.80^**^	0.89	12.22	0.34	2.97
ED^#^	19.74^**^	21.38^**^	3.31	27.79	1.37	6.02	15.12^**^	26.48^**^	2.64	30.75	1.04	7.95
GN^#^	6.62^**^	6.09^**^	1.20	14.13	0.45	1.63	7.07^**^	6.36^**^	1.56	15.21	0.46	1.60
	**2020**											
**SV**	**WS**						**WW**					
	**GCA** **(DF = 9)**	**SCA** **(DF = 15)**	**Residual** **(DF = 48)**	**X**	${\hat{\phi }}^{2}g$	${\hat{\phi }}^{2}s$	**GCA** **(DF = 9)**	**SCA** **(DF = 15)**	**Residual** **(DF = 48)**	**X**	${\hat{\phi }}^{2}g$	${\hat{\phi }}^{2}s$
GY^#^	0.46^**^	1.80^**^	0.05	1.23	0.03	0.58	1.65^**^	6.81^**^	0.15	2.87	0.12	2.22
PE^#^	62.64^**^	17.21^**^	6.14	24.67	4.71	3.69	75.94^**^	15.11^ns^	8.11	26.16	5.65	2.33
GN^#^	123.75^**^	115.33^**^	14.39	24.48	9.11	33.65	124.11^**^	86.15^**^	6.93	27.60	9.77	26.41
EL^#^	6.78^**^	13.19^**^	2.05	11.67	0.39	3.71	4.62^**^	7.50^**^	1.17	12.54	0.29	2.11
ED^#^	21.88^**^	35.98^**^	5.45	30.74	1.37	10.18	11.99^**^	29.13^**^	2.67	34.37	0.78	8.82
GN^#^	3.64^*^	4.35^**^	1.46	12.54	0.18	0.96	4.91^**^	6.27^**^	1.27	14.87	0.30	1.67

*SV, sources of variation; GCA, general combining ability; SCA, specific combining ability; WC, water conditions; DF, degrees of freedom*;

# *mean squares; X, average; GY, grain yield (t ha^−1^); PE, popping expansion (g ml^−1^); GN, grain number per row (unit); EL, ear length (cm); ED, ear diameter (mm); GW, 100-grain weight (g)*.

ns *Not significant at a 5% probability level in the F-test*.

*,** *Significant at 5 and 1% probability levels in the F-test, respectively*.

${\hat{\phi }}^{2}g$, *quadratic component associated with GCA*; ${\hat{\phi }}^{2}s$, *quadratic component associated with SCA*.

### Water Limiting Impact on Agronomic Traits Evaluated in Popcorn

**Figure 1:**
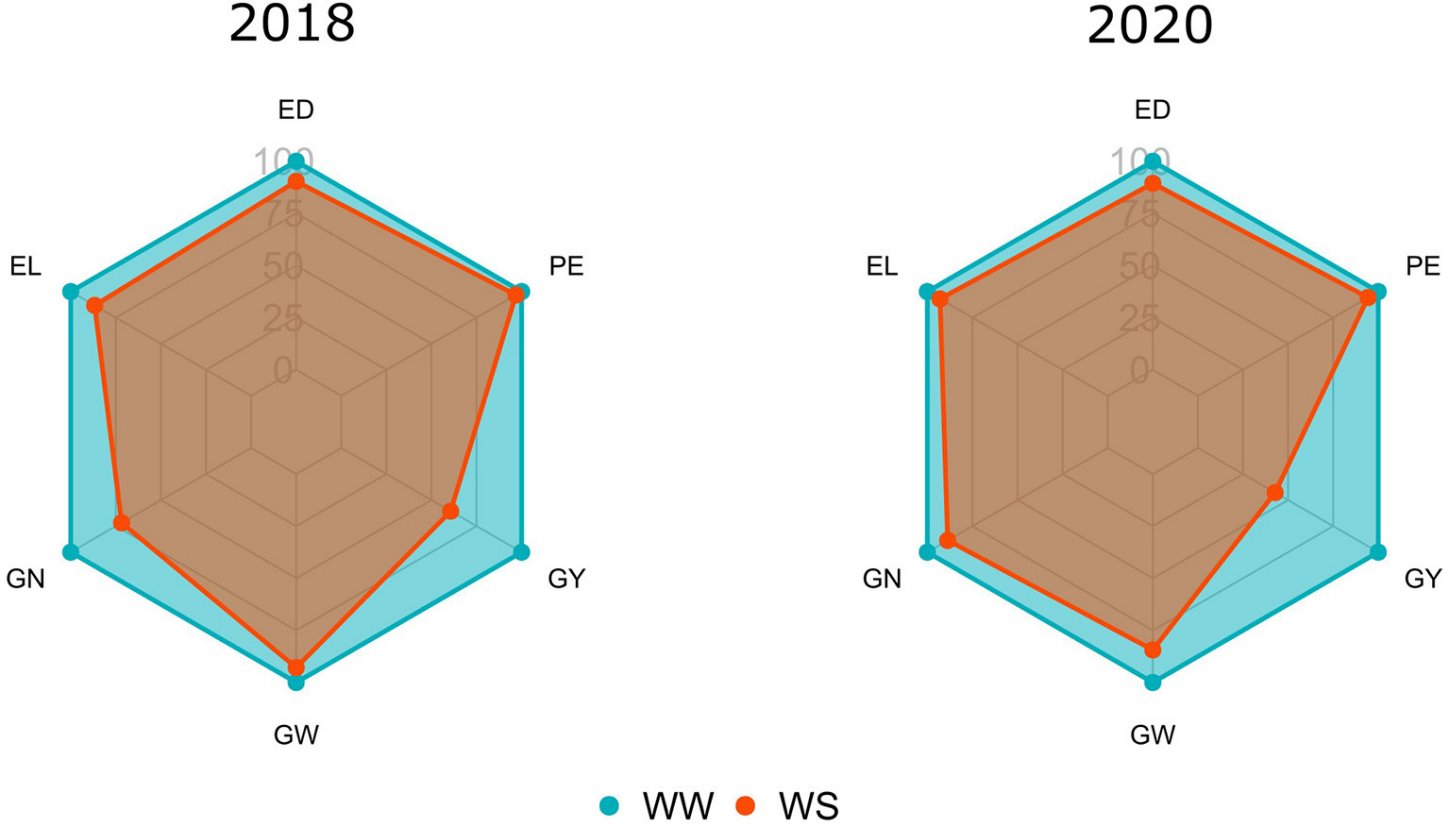
Relative percentages of the means evaluated under water stress (WS) in relation to the well-watered (WW) condition for agronomic traits [GY, grain yield; PE, popping expansion; GN, grain number per row (unit); EL, ear length; ED, ear diameter; GW, 100-grain weight] evaluated in popcorn genotypes during crop seasons 2018 and 2020.

Comparing both WCs in CS 2018, significant reductions (>15%) occurred in GY (39%) and GN (28%). In the same CS, reduced losses were observed for PE, EL, ED, and GW, with estimated values of 3.21, 13.27, 9.65, and 7.15%, respectively (Figure 1). However, when comparing WS with WW conditions in 2020, it appeared that significant reductions (>15%) occurred in GY (57%) and GW (16%). In this CS, reduced losses were observed for PE, GN, EL, and ED, with percentages of 6, 11, 7, and 10%, respectively (Figure 1).

### Values of GCA in Different WC and CS for Agronomic Traits Evaluated in Popcorn

**Figure 2:**
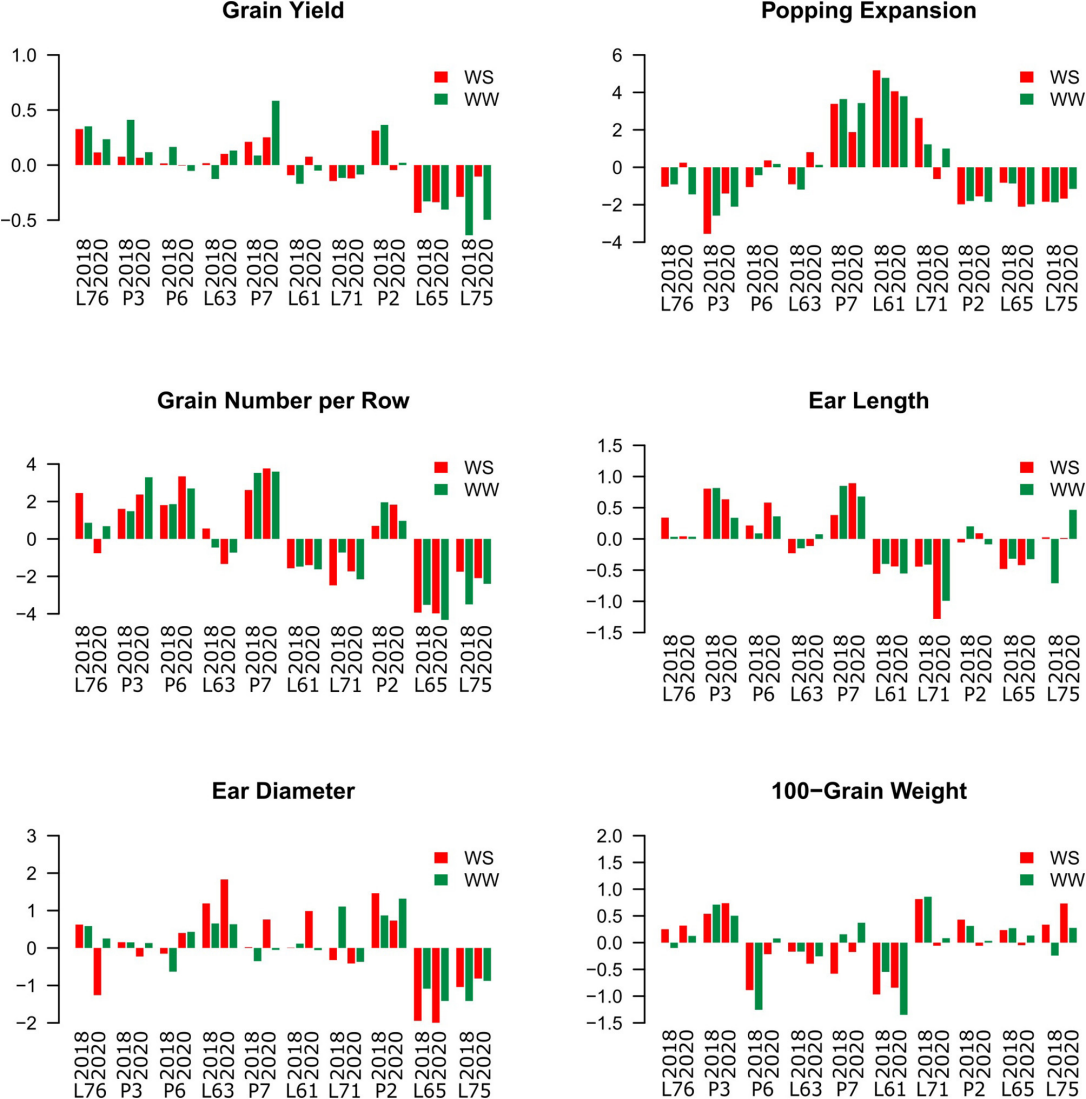
Estimation of the general combining ability (GCA) effects of popcorn inbred lines for the agronomic-assessed traits under two water conditions (WCs) and crop seasons in 2018 and 2020. Water stress (WS) is represented by red bars and the WW condition is represented by dark-green bars.

Regardless of CS and WC, the L76, P3, and P7 inbred lines showed positive estimates for GCA effects, while L71, L65, and L75 expressed negative estimations (Figure 2).

The lines P7 and L61 showed positive estimates for PE, but P3, P2, L65, and L75 expressed negative estimates (Figure 2). In particular, the P7 progenitor showed the highest GCA (3.39 in 2018 and 1.88 in 2020 under WS conditions; 3.65 in 2018 and 3.43 in 2020 under WW conditions). The L61 progenitor also had GCAs of 5.18 in 2018 and 4.06 in 2020 under WS conditions and 4.78 in 2018 and 3.79 in 2020 under WW conditions.

The P3, P6, P7, and P2 progenitors also had positive estimates for the GCA of GN, while the L61, L71, L65, and L75 progenitors showed negative estimates. Positive GCA estimates were observed for EL in the L76, P3, P6, and P7 progenitors and negative ones for L61, L71, and L65 in both WCs and CSs. The L63 and P2 progenitors showed positive estimates for ED, while L65 and L75 showed negative estimates for the same trait. Only the P3 progenitor expressed a positive GCA estimate for GW, whereas L63 and L61 progenitors had negative estimates (Figure 2).

### Values of SCA in Different WC and CS for Agronomic Traits Evaluated in Popcorn

**Figure 3:**
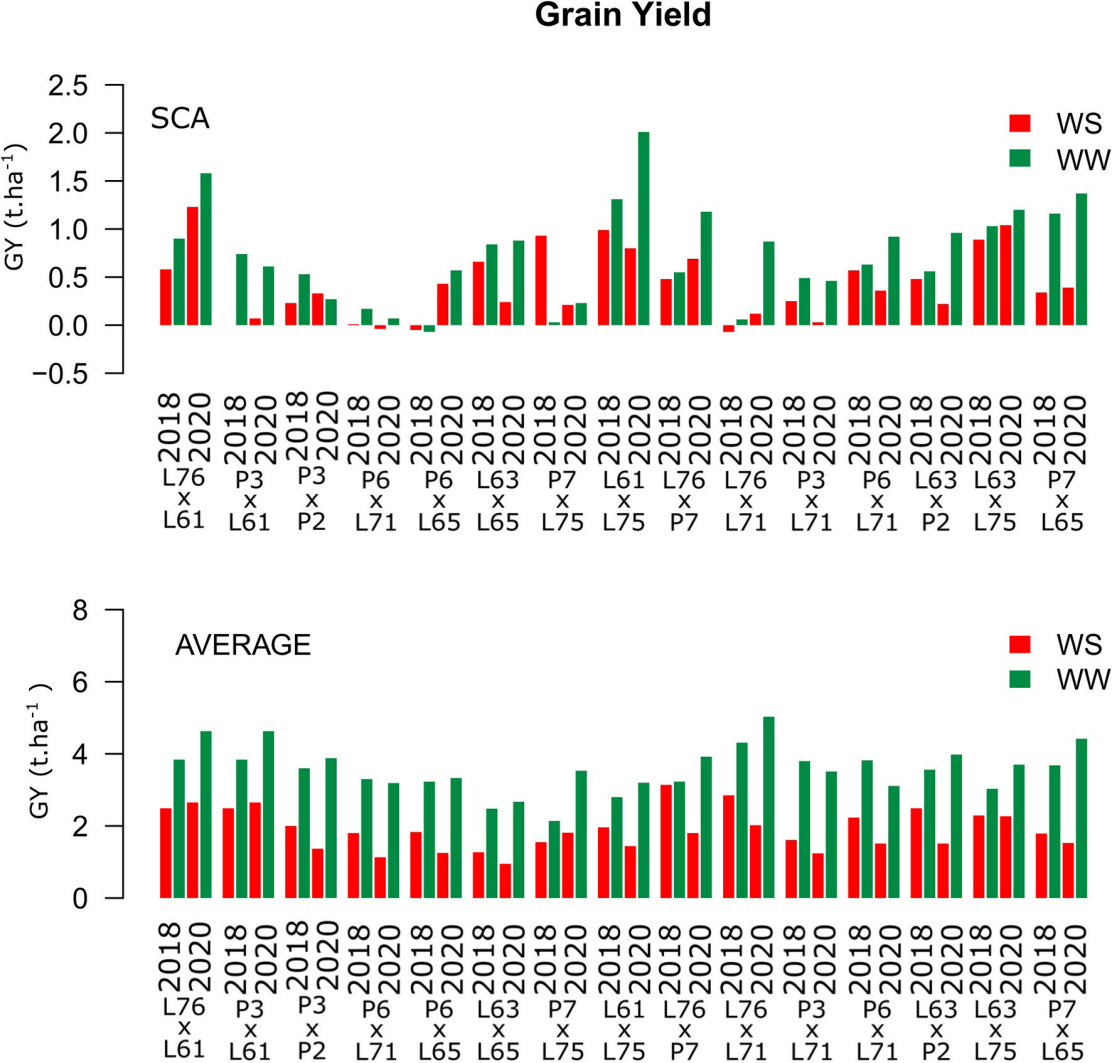
Estimation of the specific combining ability (SCA) and GY (t ha^−1^) mean values for popcorn hybrids evaluated under two WCs and crop seasons in 2018 and 2020. WS is represented by red bars and the WW condition is represented by dark-green bars.

Grain yield showed high positive estimates for SCA in the L76 × L61, L76 × L71, and L63 × L75 combinations across WCs (Figure 3). Above average mean values corresponded to the L76 × L61, P3 × L61, and L76 × L71 combinations. The best hybrids for GY were L76 × P7 (3.14 t ha^−1^) and L76 × L61 (2.65 t ha^−1^) under WS conditions in the CSs of 2018 and 2020, respectively, and L76 × L71 under WW conditions for both CSs (4.31 t ha^−1^ in 2018 and 5.03 t ha^−1^ in 2020) (Figure 3).

**Figure 4:**
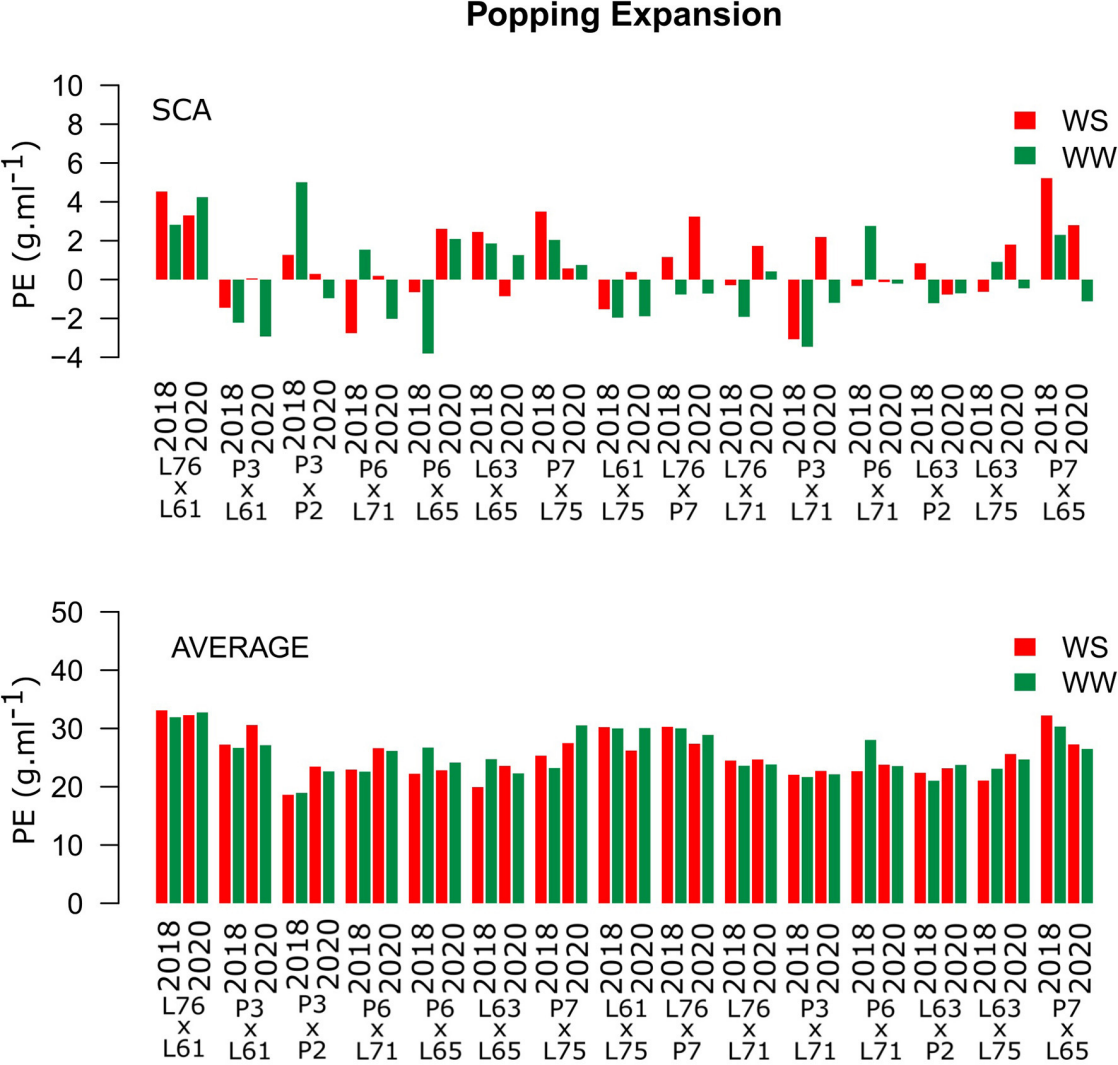
Estimation of the SCA and PE (g ml^−1^) mean values for popcorn hybrids evaluated under two WCs and crop seasons in 2018 and 2020. WS is represented by red bars and the WW condition is represented by dark-green bars.

High and positive PE SCA estimates were observed only for the L76 × L61 combination (Figure 4). The same hybrid showed the highest experimental means in both CSs and under different WCs, with estimates of 31.11 and 32.28 g ml^−1^ under WS conditions in 2018 and 2020, respectively, and 31.93 and 32.75 g ml^−1^ under WW conditions in 2018 and 2020 (Figure 4).

**Figure 5:**
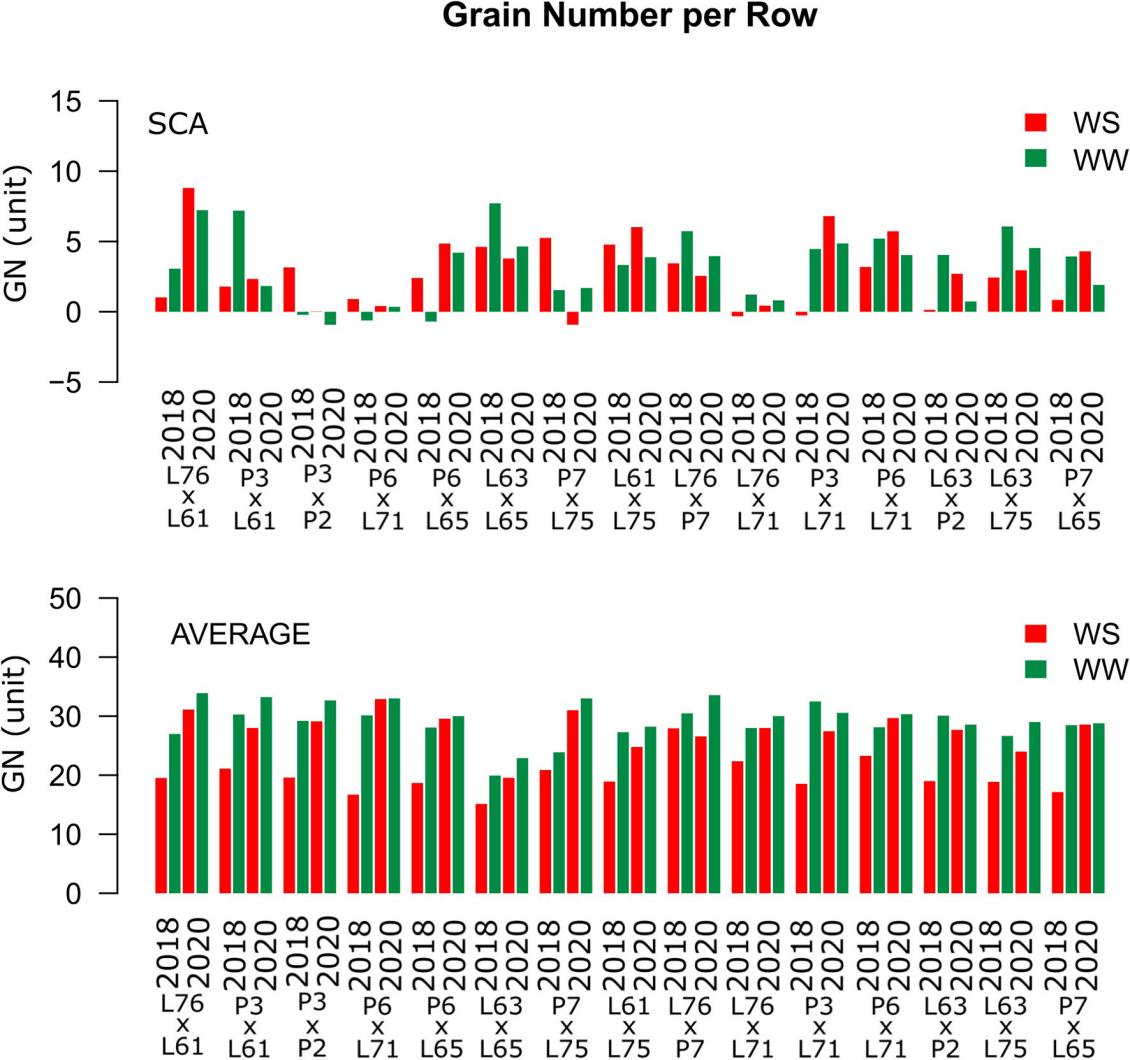
Estimation of the SCA and GN mean values for popcorn hybrids evaluated under two WCs and crop seasons in 2018 and 2020. WS is represented by red bars and the WW is represented by dark-green bars.

**Figure 6:**
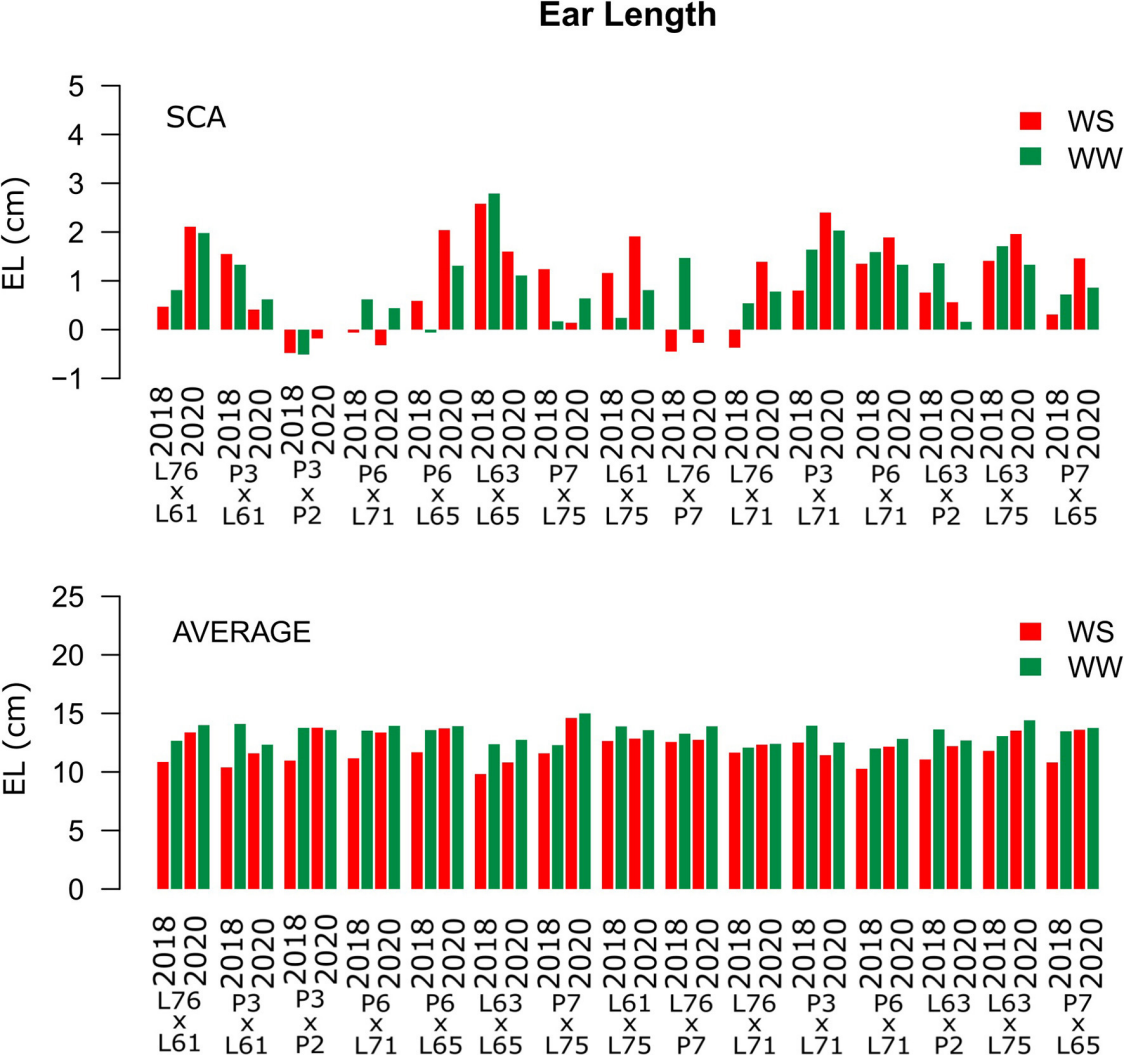
Estimation of the SCA and EL (cm) mean values for popcorn hybrids evaluated under two WCs and crop seasons in 2018 and 2020. WS is represented by red bars and the WW condition represented by dark-green bars.

**Figure 7:**
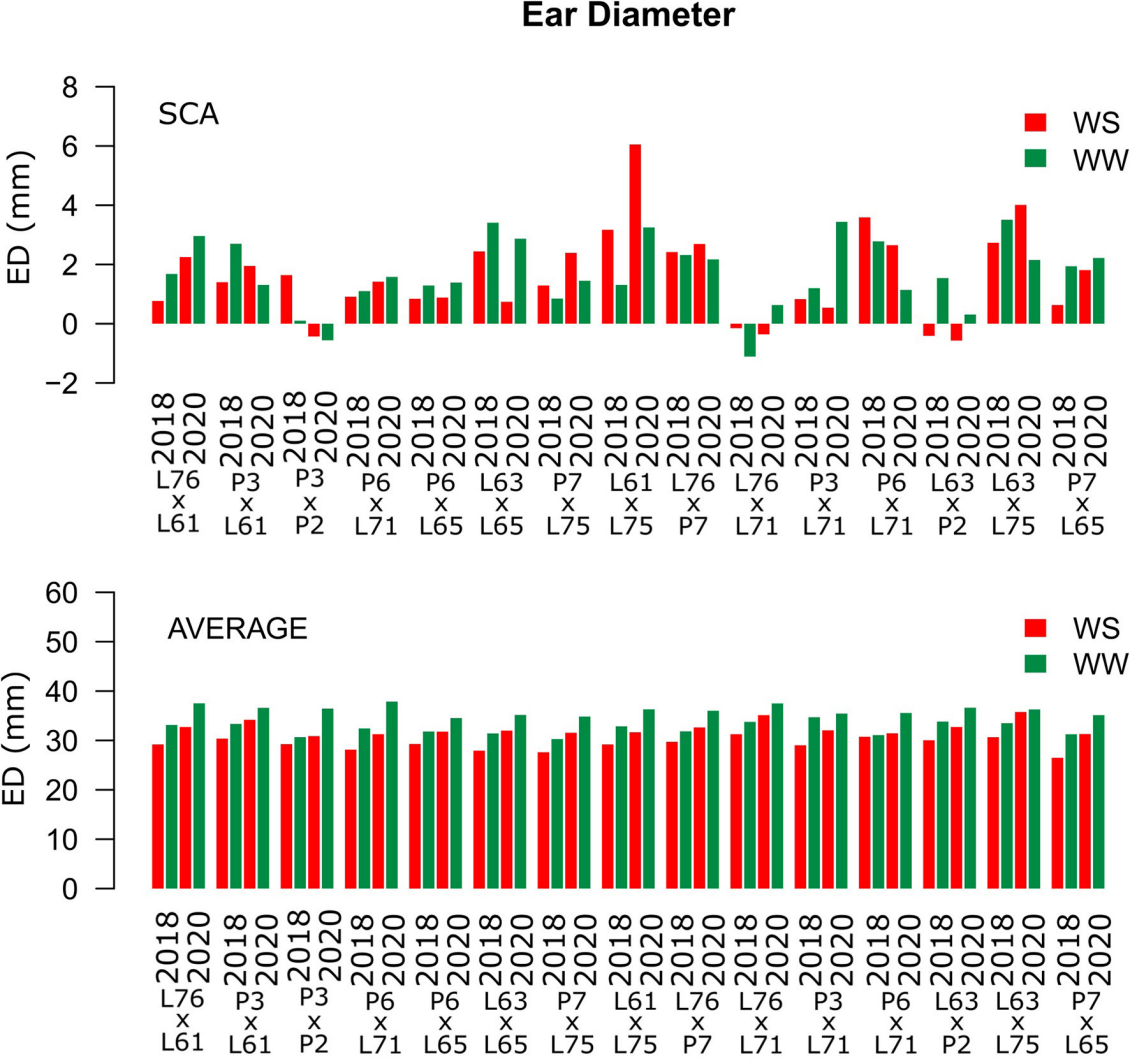
Estimation of the SCA and ED (mm) mean values for popcorn hybrids evaluated under two WCs and crop seasons in 2018 and 2020. WS is represented by red bars and the WW condition is represented by dark-green bars.

**Figure 8:**
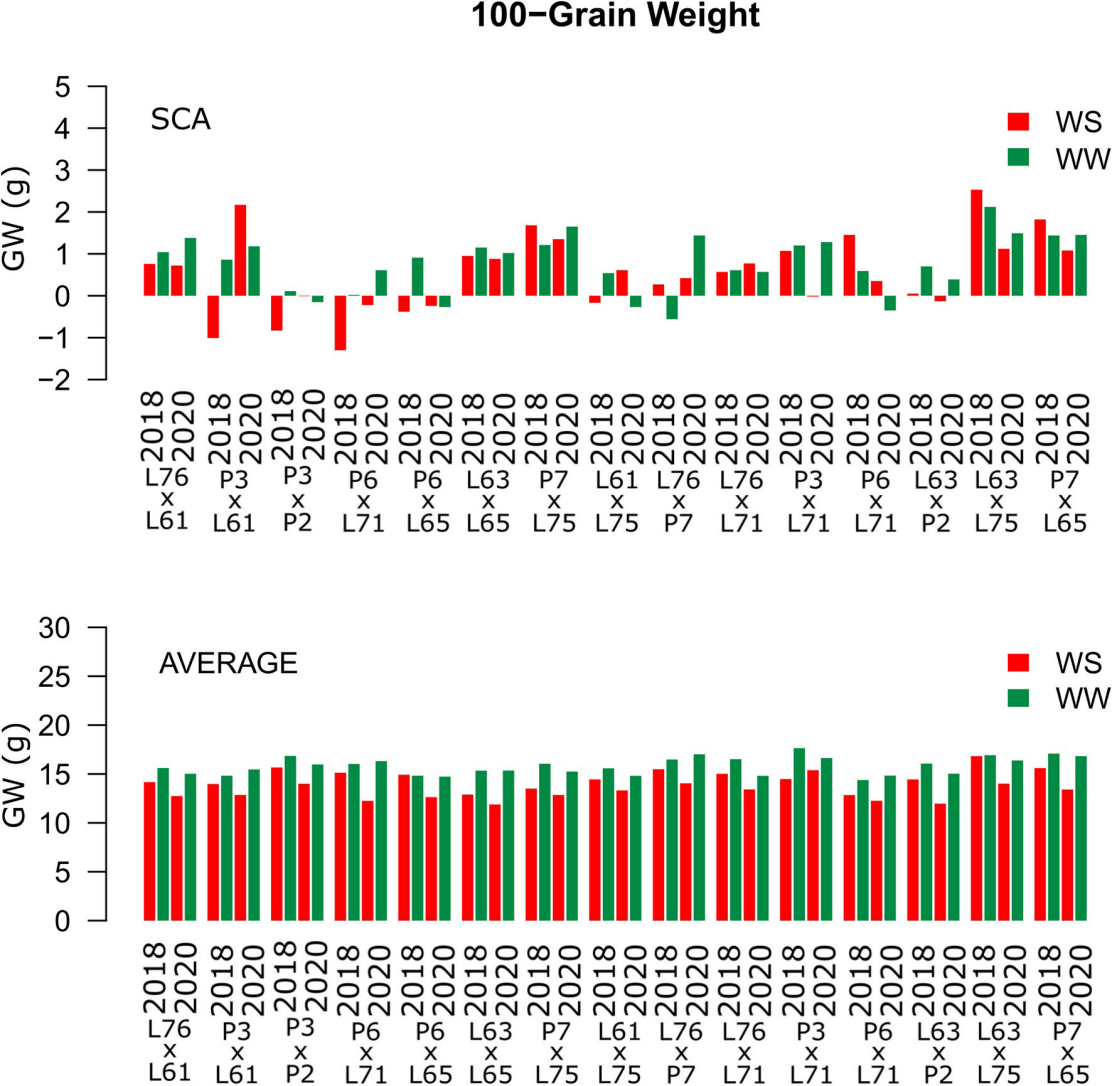
Estimation of the SCA and 100-GW (g) mean values for popcorn hybrids evaluated under two WCs and crop seasons in 2018 and 2020. WS is represented by red bars and the WW condition is represented by dark-green bars.

Considering both CS and WC, the highest SCA estimates for GN were observed for the L61 × L75 and P6 × L65 combinations (Figure 5). For EL, the L61 × L75, P6 × L71, P6 × L65, and L63 × L75 hybrids showed top performance (Figure 6), while, for ED, the superior hybrids were P3 × L61 and L63 × L75 (Figure 7). Lastly, the best combinations for GW were L61 × L75, L76 × P7, L63 × L75, and P7 × L65 (Figure 8). The largest experiment means were observed for hybrid P3 × L61 for GN (Figure 5); P6 × L65 for EL (Figure 6); P6 × L61, L76 × L71, L63 × P2, and L63 × L75 for ED (Figure 7); P3 × P2, L76 × P7, L63 × L75, and P7 × L65 for GW traits (Figure 8).

### Direct and Indirect Effects of Explanatory Variables on GY and PE Under WS and WW Conditions and Crop Seasons in 2018 and 2020

The path coefficients explained at least 89% (*R*^2^ = 0.89) of the variations in GY, with a residual effect of <33% regardless of CS and WC (Table 6). The estimated reliability of the path coefficients was also warranted by the collinearity (values considered moderate or low) according to the classification of Christensen and Montgomery (1981). In CS 2018, either under WS or WW conditions, we observed that some traits had significant total effects (*r*_*g*_) on GY as follows: GN (0.97 under WS and WW), EL (0.83 under WS and 0.88 under WW), ED (0.93 under WS and 0.88 under WW), and GW (0.54 under WS and 0.7 under WW) (Table 6). Among them, only GN had a high direct effect (0.6 under WS and 0.98 under WW) on GY (Table 6).

Table 6Path analysis of the partitioning of genotypic correlations into components of direct and indirect effects, a collinearity test, coefficient of determination, and residual effect obtained between GY and agronomic traits evaluated in popcorn genotypes under WS and WW conditions in the crop seasons of 2018 and 2020.

**Table:** 

	**PE**				**GN**				**EL**				**ED**				**GW**			
**Traits/ effect**	**2018**		**2020**		**2018**		**2020**		**2018**		**2020**		**2018**		**2020**		**2018**		**2020**	
	**WS**	**WW**	**WS**	**WW**	**WS**	**WW**	**WS**	**WW**	**WS**	**WW**	**WS**	**WW**	**WS**	**WW**	**WS**	**WW**	**WS**	**WW**	**WS**	**WW**
Direct on GY	0.14	−0.02	0.42	0.31	0.60	0.98	0.01	−0.14	0.11	−0.41	0.14	−0.03	0.26	−0.03	0.34	0.78	0.17	0.43	0.39	0.41
Indirect *via* PE	-	-	-	-	0.01	0.00	0.17	0.03	−0.01	0.00	0.18	−0.01	0.00	0.00	0.24	−0.03	−0.01	0.00	0.01	−0.09
Indirect *via* GN	0.03	0.11	0.00	−0.01	-	-	-	-	0.47	0.99	0.01	−0.11	0.53	0.99	0.01	−0.12	0.19	0.99	0.00	−0.10
Indirect *via* EL	−0.01	−0.03	0.06	0.00	0.08	−0.28	0.13	−0.02	-	-	-	-	0.07	−0.42	0.09	−0.02	0.06	−0.69	0.10	−0.02
Indirect *via* ED	−0.01	−0.01	0.19	−0.08	0.23	−0.01	0.25	0.66	0.17	−0.02	0.22	0.63	-	-	-	-	0.12	−0.04	0.15	0.54
Indirect *via* GW	−0.01	−0.03	0.01	−0.12	0.05	0.28	0.23	0.30	0.10	0.31	0.28	0.34	0.08	0.34	0.18	0.28	-	-	-	-
Total effects	0.15	0.02	0.69^**^	0.11	0.97^**^	0.97^**^	0.79^**^	0.83^**^	0.83^**^	0.88^**^	0.83^**^	0.82^**^	0.93^**^	0.88^**^	0.85^**^	0.89^**^	0.54^**^	0.70^**^	0.66^**^	0.73^**^

**Table:** 

**CS / WC**	**2018—WS**	**2018—WW**	**2020—WS**	**2020—WW**
*R* ^2^	0.99^#^	0.99^#^	0.96^#^	0.89^#^
Residual effect	0.0	0.0	0.2	0.33
Collinearity	257.72	644.47	155.51	98.44

** *Significant at 1% probability level in the F-test. R^2^: # indicate significance at a 1% probability level in the F-test; WS, water stress; WW, well-watered; CS, crop seasons; WC, water condition; GY, grain yield; PE, popping expansion; GN, grain number per row; EL, ear length; ED, ear diameter; GW, 100-grain weight*.

In CS 2020, all evaluated traits showed significant *r*_*g*_ in relation to GY, except PE under WW conditions (Table 6), which also displayed high direct effects on GY in the same CS under WS conditions, but the indirect relationship had a low magnitude. Likewise, ED and GW showed high direct effects on GY under both WCs in CS 2020. They were also the most important indirect effects on other agronomic traits, except for PE (Table 6).

We found significant path coefficient estimations for PE only under WS conditions in CS 2020, supported by a *R*^2^ = 0.85 value and a collinearity of 155.51, which were considered moderate. In this sense, there were no variables with significant *r*_*g*_ under both WC conditions and in CS 2018 (Table 7). The same result was also observed for PE under WW in CS 2020. However, in the same CS and under WS, GY (0.69), GN (0.41), EL (0.42), and ED (0.57) had significant *r*_*g*_ in relation to PE (Table 6). Among them, only GY had a high direct effect (0.99) on PE. The GN, EL, and ED, whose direct effects were low or negative on PE, had significant indirect effects on GY (Table 7).

Table 7Path analysis of the partitioning of genotypic correlations into components of direct and indirect effects, a collinearity test, coefficient of determination, and residual effect obtained between PE and agronomic traits evaluated in popcorn genotypes under WS and WW conditions in the crop seasons of 2018 and 2020.

**Table:** 

	**GY**				**GN**				**EL**				**ED**				**GW**			
**Traits/effect**	**2018**		**2020**		**2018**		**2020**		**2018**		**2020**		**2018**		**2020**		**2018**		**2020**	
	**WS**	**WW**	**WS**	**WW**	**WS**	**WW**	**WS**	**WW**	**WS**	**WW**	**WS**	**WW**	**WS**	**WW**	**WS**	**WW**	**WS**	**WW**	**WS**	**WW**
Direct on PE	−0.99	0.35	0.99	0.99	0.80	−0.99	−0.10	0.68	0.01	0.78	0.02	0.47	0.18	0.43	−0.19	−0.99	0.37	−0.55	−0.76	−0.99
Indirect *via* GY	-	-	-	-	−0.99	0.33	0.99	0.85	−0.99	0.31	0.99	0.87	−0.99	0.32	0.99	0.82	−0.99	0.26	0.99	0.68
Indirect *via* GN	0.82	−0.99	−0.04	0.56	-	-	-	-	0.63	−0.99	−0.08	0.58	0.68	−0.90	−0.06	0.53	0.43	−0.71	−0.08	0.46
Indirect *via* EL	0.01	0.69	0.02	0.37	0.01	0.74	0.02	0.37	-	-	-	-	0.01	0.65	0.02	0.33	0.01	0.60	0.02	0.35
Indirect *via* ED	0.18	0.36	−0.10	−0.98	0.16	0.33	−0.18	−0.99	0.13	0.32	−0.15	−0.99	-	-	-	-	0.14	0.34	−0.15	−0.80
Indirect *via* GW	0.13	−0.39	−0.18	−0.83	0.07	−0.36	−0.32	−0.84	0.13	−0.40	−0.36	−0.94	0.10	−0.43	−0.19	−0.78	-	-	-	-
Total effects	0.15	0.02	0.69^**^	0.11	0.05	0.05	0.41^**^	0.08	−0.10	0.02	0.42^**^	−0.02	−0.03	0.07	0.57^**^	−0.10	−0.04	−0.07	0.02	0.30

**Table:** 

**CS / WC**	**2018—WS**	**2018—WW**	**2020—WS**	**2020—WW**
*R* ^2^	0.02	0.04	0.85^#^	0.62^#^
Residual effect	0.99	0.98	0.39	0.62
Collinearity	257.72	644.47	155.51	98.44

** *Significant at 1% probability level in the F-test. R^2^: #, indicate significance at a 1% probability level in the F-test; WS, water stress; WW, well-watered; CS, crop seasons; WC, water condition; GY, grain yield; PE, popping expansion; GN, grain number per row; EL, ear length; ED, ear diameter; GW, 100-grain weight*.

While GY was considered the main trait, regardless of CS, the comparison of the total effects of the explanatory agronomic traits under WW and WS indicated that these parameters had similar patterns. The GN, EL, ED, and GW traits had similar values for the total effects and were in the same direction (Table 7). The same patterns were not observed when PE was considered as the main trait mainly due to the absence of significant total effects (Table 7).

## Discussion

### Genetic Variability for Agronomic Traits Evaluated in Popcorn in Different WCs and CSs

There was genetic variability in the traits of greatest economic interest for popcorn (GY and PE). Stopping irrigation pre-anthesis was sufficient to differentiate WCs. As a result, there were significant differences between WS and WW for GY and PE. Indeed, pre-anthesis drought stress is a well-known practice for genotypic evaluation in maize under drought conditions (Carena et al., 2009; Araus et al., 2010; Romano et al., 2011; Cairns et al., 2012; Zia et al., 2013; Adebayo et al., 2014; Kamphorst et al., 2019, 2020a). Through reduced irrigation, plants can express possible adaptations to water-limiting conditions in a timely manner, which can be evaluated in final productivity (Romano et al., 2011; Cairns et al., 2012). At this phenological stage, water stress can compromise pollen viability, zygote formation, and grain filling, which are biological processes that are highly sensitive to soil water limitation (Zinselmeier et al., 1995), decreasing GY.

Owing to CS and WC significant effects and significant interactions with genotypes (G), the differential responses of popcorn inbred lines and hybrids for GY were expected. In plant breeding, interactions of this nature influence the recommendation of cultivars for specific environmental conditions and genetic gain (Hallauer et al., 2010). Likewise, PE showed a differential response across popcorn genotypes. In this case, mainly in relation to WC effect, it appears that CS effects did not show large relevance to PE as CS and CS^*^WC effects were not significant. In both cases, an alternative to achieve genetic gains could be the identification of correlated traits to be used in indirect selections, which are determinants in the expression of main traits. Interestingly, these traits did not show WC^*^G significant interactions, but were significantly and positively associated with the main traits (Kamphorst et al., 2019). The lack of significance for G^*^CS, G^*^WC, and G^*^CS^*^WC interactions in relation to GN and GW supports our assertion that, for these traits, selections under water-deficit or well-watered conditions are effective in obtaining genetic gains simultaneously in both CSs and WCs.

Indeed, the aforementioned popcorn traits have been identified to be of great importance in the literature under adequate water availability (Amaral Júnior et al., 2016; Cabral et al., 2016; de Lima et al., 2016) and drought conditions (Kamphorst et al., 2019, 2020a). However, these traits showed significant CS^*^WC interactions, which was mainly due to the specific moment when water deficits occurred with higher intensity across CSs (i.e., the fertilization period in CS 2018 and grain filling in 2020), which differentially influenced the number and weight of grains (Cairns et al., 2012). Lastly, EL and ED did not express significant interactions with WC. These traits were determined before the flowering period (Durães et al., 2004), and, therefore, had lower influence on the specific moment in the reproductive period when water deficits occur with greater intensity. However, the influence of WC seems to be more relevant for EL and ED, given that G^*^CS was the only significant interaction for these traits.

### Genetic Effects of WC in Different CSs for Agronomic Traits Evaluated in Popcorn

Under water-deficit conditions, knowing the patterns of important agronomic traits and secondary traits is essential for the development of more efficient strategies in the early stages of plant-breeding programs (Derera et al., 2008). Thus, our results shed light on the influence of WCs on GCA and SCA estimates for all traits in both CSs. Moreover, given that GCA^*^WC and SCA^*^WC for GY and SCA^*^WC for PE had significant effects, a differentiated response of genotypes under different WCs and in both CSs can be expected. The significant GCA interaction for GY indicated that there was no consistency between the additive effects observed in each WC for this trait. It means that a progenitor with greater additive effects in one condition may not have the same superiority in another condition. However, the component ${\hat{\phi }}^{2}gwc$ variation which was shown to be <2% of the total variation observed certainly will not change the responses of the progenitors for GY.

The significant SCA interaction for GY and PE indicated an alteration in allelic complementation among progenitors in loci with some degree of dominance for these two traits. In this case, the component ${\hat{\phi }}^{2}swc$ displayed a variation of 14% in GY and 18% in PE on average, which could lead to alterations in SCA for GY and PE estimations. It happens because alleles that drive the expression of a specific trait under an environmental condition are partially different from alleles that control that same trait under a different condition (Hao et al., 2010; Lu et al., 2011). In other words, traits under water deficits contain genes other than those expressed under well-watered conditions; therefore, some genes may have been expressed and/or silenced (Hao et al., 2010; Lu et al., 2011; Rahman et al., 2011). However, the variation of the estimates ${\hat{\phi }}^{2}g$ and ${\hat{\phi }}^{2}s$ summed represented more than 79% of the variation in GY and 74% of the variation in PE. Thus, it will permit, at least in part, the selection of progenitors and independent crosses regardless of WC.

For the GY in each CS, the magnitudes of the quadratic components showed the greatest importance of dominance gene effects $({\hat{\phi }}^{2}s$), representing 76 (2018) and 75% (2020) of the total variation. Therefore, our results indicate that breeding methods focused on exploiting heterotic effects would be the best alternatives to provide superior gains regardless of WC. On the other hand, the greater importance of additive effects $({\hat{\phi }}^{2}g$) was evident for PE, with an explanation of 51 (2018) and 54% of the total variation. In this case, the use of progenitors in intrapopulation breeding programs is the best alternative to achieve gains through selection processes (Pereira and Amaral Júnior, 2001; Ribeiro et al., 2016). Thus, inbred lines obtained from populations with advanced intrapopulation selection cycles are very likely to concentrate additive alleles favorable to PE. These inbred lines can be crossed to explore the heterosis of other traits and thus provide effective gains for all economically important traits (Ribeiro et al., 2016; Lima et al., 2019).

The GN, EL, ED, and GW traits, which are important co-variables of the most important traits for popcorn, also had the greatest importance for dominance effects $({\hat{\phi }}^{2}s$). This component represented more than 70% of the variation regardless of WC. Unlike what happened for GY and PE, it is noteworthy that the ${\hat{\phi }}^{2}gwc$ and ${\hat{\phi }}^{2}swc$ effects were not significant. Therefore, the stable responses of genotypes for these traits under different WCs are warranted. Indeed, under well-watered conditions, dominance effects are reported as the main components of GY expression in popcorn, as are additive effects for PE (Pereira and Amaral Júnior, 2001; Schwantes et al., 2017; Mafra et al., 2018; Coan et al., 2019; Schmitt et al., 2019).

For maize under drought conditions, both non-additive (Derera et al., 2008; Adebayo et al., 2014) and additive effects were observed (Chapman and Edmeades, 1999; Betrán et al., 2003; Dhliwayo et al., 2009). The prevalence of the dominance effects for GY and additive effects for PE was observed in popcorn under soil nitrogen (N)-limited conditions (Santos et al., 2019, 2020) and phosphorus (P)-limited conditions (Gerhardt et al., 2019). When evaluating popcorn genotypes under water-deficit conditions, Lima et al. (2019) verified that dominance effects prevailed for GY while the same was true for additive effects for PE. Since there are differences between WCs and a significant interaction between GCA and SCA under WCs, selection programs must be conducted for each WC in each CS.

### Combining Ability for Agronomic Traits Evaluated in Popcorn Under WC in Each CS

Despite the significant interactions of GCA and SCA related to WCs, combining abilities were similar in both CSs and WCs (Table 4), as the estimates of the magnitude of these interactions had a reduced contribution to the total variation. This supports our proposal of the best progenitors and hybrids. Regarding PE, the estimates of GCA effects on the P7 and L61 inbred lines showed that they were the most promising candidates, as the effects revealed the highest positive magnitudes of g_i_ in both CSs and WCs. As PE is negatively correlated with GY, inbred lines with high GCA values for PE and low CGC values for GY or *vice versa* are commonly obtained (Cabral et al., 2016; de Lima et al., 2016; Mafra et al., 2018; Gerhardt et al., 2019) as we observed for L61. However, P7 had high values for both traits, which makes it of great importance for breeding programs when simultaneously exploring the potential for GY and PE. The estimates of GCA effects for GY allowed the identification of the L76, P3, and P7 inbred lines as the most promising ones given the highest positive magnitudes of g_i_ under WS and WW conditions regardless of CS. Although g_i_ effect is not the most important for this trait, these inbred lines are more likely to generate superior hybrids in crosses with different inbred lines.

Regarding SCA effects for GY in both CS and WCs, the combinations with the highest estimates were L76 × L61, L76 × L71, and L63 × L75. For the first two hybrids, we observed that these combinations had at least one progenitor (L76) with a high GCA. In addition to the high SCA effects, the same two hybrids had above average GY values. In the same sense, the L76 × L61 hybrid also showed the highest SCA for PE and the highest average estimated under each WS and CS. Therefore, this hybrid is a promising genotype, considering the traits of main economic importance for popcorn crops.

### Water Limitation Impacts on Agronomic Traits Evaluated in Popcorn

Grain yield was the trait that was most affected by soil water limitation regardless of CS. The WS applied between the pre-anthesis and grain-filling periods reduced the yield covariates to a lesser extent. However, the sum of these effects drastically reduced the main variable (GY). Yet, it is clear that, in CS 2018, the most affected covariate was GN (28.2%) and GW in CS 2020 (15.63%).

The reduction in the number of grains produced per area under drought conditions was identified as the trait responsible for the reduction of GY in maize (Cairns et al., 2012). Pollen viability and zygote formation are processes sensitive to WS (Zinselmeier et al., 1995), which reduces the number of grains produced. This may have occurred in CS 2018, where the effects of water deficits appeared earlier (at a stage before anthesis), impacting the expression of GN. However, WS effects occurred later in CS 2020, and the main negative effect of water deficit was perceived in 100-GW and, therefore, GY.

Soil water limitation exerted little impact on PE in both CSs. However, proportional reductions in both WCs were greater in 2020 (5.68%). In CS 2020, water deficits strongly affected grain filling and, possibly, the quality of the grain. In fact, Kamphorst et al. (2020a), in an evaluation of popcorn inbred lines, reported reductions of 29.31 and 9.66% when comparing WS with WW. Yet, Lima et al. (2019) observed a reduction of 9.08% when popcorn inbred lines and hybrids were evaluated. The expansion process is associated with moisture presence in the starch granules of the grain. When heated (≈180°C), the moisture exerts pressure on the pericarp, with rupturing subsequently exposing the endosperm (Silva et al., 1993). This means that water shortages during grain formation can affect physicochemical properties and, therefore, the capacity of the grain to expand. Moreover, no morphological or chemical traits that could explain this phenomenon have been recorded to date.

### Direct and Indirect Effects of the Explanatory Variables on the Main Variables (GY and PE) Under WS and WW Conditions in Different Crop Seasons

Correlation coefficients are very useful for quantifying the magnitude and association of factors in determining complex traits but still do not give the exact importance of direct and indirect effects (Cruz et al., 2012). Thus, path analyses were performed to know the direct and indirect effects of the most important secondary agronomic traits, subsequently explaining the highest GY and PE values. In addition to knowing the cause-and-effect relationship in GY and PE, this approach aimed to find an alternative for overcoming the influence of the G^*^CS, G^*^WC, and G^*^CS^*^WC significant interactions and that of GCA^*^WC and SCA^*^WC that can hinder plant selection and cultivar recommendation. This could be an alternative, considering that GN, EL, ED, and GW traits did not present significant interactions for G^*^WC and G^*^CS^*^WC or for CGA^*^WC and SCA^*^WC.

In this context, when comparing the total effects (*r*_*g*_) of GN, EL, ED, and GW traits between WS and WW conditions, there was a similarity in the responses (significance and direction) for GY. In fact, GN, ED, and GW traits are suitable for popcorn plant selection as they contribute to increasing GY, given the significant and positive correlations between them, both under well-watered (Cabral et al., 2016; de Lima et al., 2016) and drought conditions (Kamphorst et al., 2018b, 2020a). However, the main cause-and-effect factor on GY changed across CSs but not WCs. Thus, we highlighted that GN was the cause of the greatest direct and indirect effects on GY in CS 2018, whereas ED was the trait with the greatest direct and indirect influence on GY in CS 2020 regardless of WC.

As contributions to popcorn-breeding programs, which aim to increase GY under WW and WS conditions, even with significant G^*^CS interaction, GN and ED traits are recommended to support selection.

When PE was considered as the main trait, it was found that there was no similarity of response (significance and direction) across WSs or CSs. This may indicate the absence of a cause-and-effect relationship of the explanatory variables for PE. Only in CS 2020 and under the WS condition did GY have a high direct effect (*r*_*g*_) on PE and indirect effects on GN, EL, and ED, having the expressive action of the GY trait. Positive, significant, and high associations between PE and GY are rare, but they have already been reported by Kamphorst et al. (2019) in drought conditions. Recently, Parsons et al. (2020) described a positive association between agronomic (ear length, number of kernel rows per ear, ear weight, 100-grain weight, and kernel size) and popping traits (kernel vitreousness, popability, and expansion volume). Alternatives to overcome this challenging trend of negative association between agronomic and popping traits were suggested by Amaral Júnior et al. (2016), given the development of the supertrait expanded popcorn volume (m^3^ ha^−1^), obtained by multiplying GY (kg ha^−1^) and PE (ml g^−1^), which allowed simultaneous gains in these two traits. Likewise, Parsons et al. (2020, 2021) developed a selection index based on potential genetic value built with the sum of the phenotypic value and the economic weight of yield and grain quality traits of importance for the crop. As prospects, both alternatives should be tested in further research for the selection of superior genotypes and/or for simultaneous genetic gains for GY and PE. In addition, working with genetically broad-based germplasms will always increase the chances for successful multi-trait selections.

## Conclusions

Both additive and dominance effects were relevant to determining the assessed agronomic traits under water-deficit and well-watered conditions. However, in both water conditions, dominance effects were more important for the expression of grain yield, grain number per row, ear length, ear diameter, and 100-grain weight. Meanwhile, for popping expansion, additive effects prevailed. Owing to the null estimates of ${\hat{\phi }}^{2}gwc\;$and ${\hat{\phi }}^{2}swc\;$effects, the stable responses of genotypes in different water conditions for grain number per row, ear length, ear diameter, and 100-grain weight traits were expected.

Hybrids are recommended for plant-breeding programs, which aim for the adaptation of plants to drought conditions as genetic dominance effects drive most yield traits, especially under water-limiting conditions. However, selected progenitors must have above average popping expansion to obtain superior hybrids. We conclude that similar breeding methods applied to popcorn under well-watered conditions can be used to develop superior genotypes under water deficits.

## Data Availability Statement

The original contributions presented in the study are included in the article/Supplementary Materials; further inquiries can be directed to the corresponding author/s.

## Author Contributions

SK and VL: conceptualization. SK, AA, VL, VA, GM, PS, JL, KS, DS, RB, TS, UO, JP, DL, CC, LG, and JS: methodology and investigation. SK and VL: software. SK, AA, MC, and EC: resources. SK, AA, VL, MC, and EC: writing—original draft preparation. AA and EC: supervision. All authors contributed to the article and approved the submitted version.

## Funding

This study was financed in part by the Coordenação de Aperfeiçoamento de Pessoal de Nível Superior—Brasil (CAPES)—Finance Code 001. Funding was also provided by FAPERJ, with the project E26/201.813/2017, for SK and E26/202.761/2017 for AA.

## Conflict of Interest

The authors declare that the research was conducted in the absence of any commercial or financial relationships that could be construed as a potential conflict of interest.

## Publisher's Note

All claims expressed in this article are solely those of the authors and do not necessarily represent those of their affiliated organizations, or those of the publisher, the editors and the reviewers. Any product that may be evaluated in this article, or claim that may be made by its manufacturer, is not guaranteed or endorsed by the publisher.

## Supplementary Material

Supplementary Figure 1 Soil water potential (MPa) in days after sowing (DAS) for experiments performed during the 2018 and 2020 harvests with popcorn inbred lines in water-stressed (WS in red) and well-watered (WW in blue) conditions.

Supplementary Figure 2 Weekly average values on DAS of temperature (°C), relative humidity (%), and solar radiation (μmol m^−2^s^−1^) throughout the growing period of the experiments with popcorn inbred lines during the 2018 and 2020 crop seasons.

## Abbreviations

CS: crop seasons
DF: degrees of freedom
ED: ear diameter
EL: ear length
G: genotype
GCA: general combining ability
GN: grain number per ear
GY: grain yield
GW: 100-grain weight
MS: mean squares
PE: popping expansion
SS: sum of squares
SV: source of variation
WC: water conditions
SCA: specific combining ability.
